# Biomimetic Design of a Tendon-Driven Myoelectric Soft Hand Exoskeleton for Upper-Limb Rehabilitation

**DOI:** 10.3390/biomimetics8030317

**Published:** 2023-07-19

**Authors:** Rodrigo C. Silva, Bruno. G. Lourenço, Pedro H. F. Ulhoa, Eduardo A. F. Dias, Fransergio L. da Cunha, Cristiane P. Tonetto, Luis G. Villani, Claysson B. S. Vimieiro, Guilherme A. Lepski, Marina Monjardim, Rafhael M. Andrade

**Affiliations:** 1 Department of Mechanical Engineering, Universidade Federal do Espírito Santo, Vitória 29.075-910, Brazil; rodrigo.silva.06@edu.ufes.br (R.C.S.); bruno.lourenco@edu.ufes.br (B.G.L.); fransergio.cunha@gmail.com (F.L.d.C.); cris.tonetto@gmail.com (C.P.T.); luis.villani@ufes.br (L.G.V.); 2 Department of Electrical Engineering, Universidade Federal do Espírito Santo, Vitória 29.075-910, Brazil; pedrofabriz2000@gmail.com; 3 Graduate Program of Mechanical Engineering, Universidade Federal do Espírito Santo, Vitória 29.075-910, Brazil; eduardo.afdias@gmail.com; 4 Graduate Program of Mechanical Engineering, Universidade Federal de Minas Gerais, Belo Horizonte 31270-901, Brazil; claysson@gmail.com; 5 Departments of Neurology and Psychiatry, Medical School, Universidade de São Paulo, São Paulo 05403-010, Brazil; lepski@gmail.com; 6 Graduate Program of Animal Biology, Universidade Federal do Espírito Santo, Vitória 29.075-910, Brazil; marina.monjardim@gmail.com

**Keywords:** biomimetic design, soft hand exoskeleton, myoelectric control, rehabilitation

## Abstract

Degenerative diseases and injuries that compromise hand movement reduce individual autonomy and tend to cause financial and psychological problems to their family nucleus. To mitigate these limitations, over the past decade, hand exoskeletons have been designed to rehabilitate or enhance impaired hand movements. Although promising, these devices still have limitations, such as weight and cost. Moreover, the movements performed are not kinematically compatible with the joints, thereby reducing the achievements of the rehabilitation process. This article presents the biomimetic design of a soft hand exoskeleton actuated using artificial tendons designed to achieve low weight, volume, and cost, and to improve kinematic compatibility with the joints, comfort, and the sensitivity of the hand by allowing direct contact between the hand palm and objects. We employed two twisted string actuators and Bowden cables to move the artificial tendons and perform the grasping and opening of the hand. With this configuration, the heavy part of the system was reallocated to a test bench, allowing for a lightweight set of just 232 g attached to the arm. The system was triggered by the myoelectric signals of the biceps captured from the user’s skin to encourage the active participation of the user in the process. The device was evaluated by five healthy subjects who were asked to simulate a paralyzed hand, and manipulate different types of objects and perform grip strength. The results showed that the system was able to identify the intention of movement of the user with an accuracy of 90%, and the orthosis was able to enhance the ability of handling objects with gripping force up to 1.86 kgf.

## 1. Introduction

The functionality of the hand is an essential component for daily living activities, as it facilitates the manipulation of objects and the interaction with the surrounding environment [1]. Regrettably, upper-limb impairments following cerebrovascular accidents (strokes) and spinal cord injuries result in functional limitations of the hand and the entire upper-limb [2]. It has been estimated that nearly 15 million individuals experience strokes annually, with approximately 80% of these cases manifesting some degree of motor impairment [3]. This makes stroke one of the major causes of disability worldwide [4]. Such disabilities substantially impact the quality of life for affected individuals, frequently impeding their ability to carry out rudimentary daily tasks and generating a significant social and economic burden [5].

Numerous rehabilitative approaches have been established to assess and restore functionalities in paretic hands after a stroke [6]. It has been determined that crucial elements, such as repetition and active patient participation in therapeutic exercises, significantly impact rehabilitation outcomes [7,8,9]. Robotic rehabilitation has emerged as an innovative therapeutic modality that may offer an alternative to conventional rehabilitation methods for post-stroke patients [10]. Robotic systems offer a great capacity for executing repetitive tasks and can adjust user active participation based on the applied control strategy, making them great rehabilitation tools.

Several designs have been presented for hand rehabilitation exoskeletons, which differ in mechanical and kinematical configurations [11]. These are commonly classified into two main categories: rigid and soft exoskeletons. Rigid exoskeletons are typically composed of mechanical links and gearmotors in a parallel mechanism that closely follows the anatomy and kinematics of the human hand [12,13]. Despite their ability to provide accurate and controllable force transmission, rigid exoskeletons often suffer from drawbacks such as bulkiness, high cost, and limited adaptability to individual patient morphologies [14]. Furthermore, the lack of kinematic compatibility between the exoskeleton and the human joint can lead to high interaction torques, and injure the user or reduce the results of the rehabilitation process [15]. On the other hand, soft exoskeletons incorporate flexible materials and soft actuators, offering lightweight, comfortable, and adaptable solutions that conform to a wide range of hand sizes and shapes [16]. Even if soft exoskeletons present challenges in terms of force generation [17], recent advances in soft materials and structures, e.g., topology optimization of flexure joints, prove that enhancing the torsional stiffness of compliant mechanisms and improving control accuracy are possible [18,19].

To address these challenges, novel designs and actuation strategies for hand exoskeletons strike a balance between rigidity and flexibility, with the aim of optimizing both force generation and user comfort [20]. Tendon-driven hand exoskeletons have gained traction as they employ a network of artificial tendons, typically consisting of cables or wires, to transmit forces from remotely located actuators to the hand’s joints, reducing the overall system weight and complexity, while still providing precise force control [21]. Recent advances in the field include underactuated exoskeletons, which use a reduced number of actuators to control multiple joints, thus reducing the complexity of the system [22].

Additionally, underactuation also allows for a reduction in cost in regard to active devices, as one of the primary contributors to the high cost of these devices is the use of complex gears and intricate transmission systems, which add to the manufacturing expenses. To address these shortcomings, the use of twisted string actuators (TSA) has been explored as a cost-effective and lightweight alternative to traditional gears and motors [23]. TSAs rely on the coiling and uncoiling of flexible cables to generate motion and transmit force, providing a simple and efficient solution for tendon-driven exoskeleton designs [24]. The integration of TSA into hand exoskeleton designs has the potential to significantly reduce the overall cost and complexity of these devices, making them more accessible to a wider range of users in need of rehabilitation. An example is the ExoTen-Glove presented by Hosseini et al. [25], a hand orthosis with artificial finger extensor tendons driven by two TSA, one for a group of fingers from index to pinky finger, and one for the thumb. The design was used to generate force to resist the grasping movement performed by the user, allowing for the simulation of a virtual spring through a virtual reality environment. However, since the flexor tendons of the fingers were not implemented, the system could not provide a grasping movement.

This paper introduces a novel tendon-driven myoelectric hand exoskeleton that combines the benefits of lightweight design, cost-effectiveness, and intuitive control. Inspired by the natural functioning of human tendons, the exoskeleton employs three artificial tendons per finger, enabling independent movements and a range of grasping actions when working in unison. By intentionally leaving the palm and phalanges uncovered, the design facilitates enhanced tactile feedback and direct contact with objects, promoting a more natural and immersive rehabilitation experience for the user. Preliminary myoelectric control is also presented, and the exoskeleton’s control system is assessed in healthy subjects. This innovative approach to hand exoskeleton development attempts to overcome some of the limitations of existing designs.

## 2. Materials and Methods

### 2.1. Bioinspired Design

Design meets challenges that nature has already solved with practical and efficient solutions through thousands of years of evolution and natural selection. As a result, nature has been the main source of inspiration over the centuries in the development of agriculture, architecture, construction, energy, transport, and technology. Then, the proposed kinematics of the hand exoskeleton were inspired by the anatomy of the hand [26]. Unlike most existing devices, the choice of biodesign, mimicking the origin and structures of human tendons, allowed the reproduction of the natural movement of joints, fingers, and hands.

**Figure 1:**
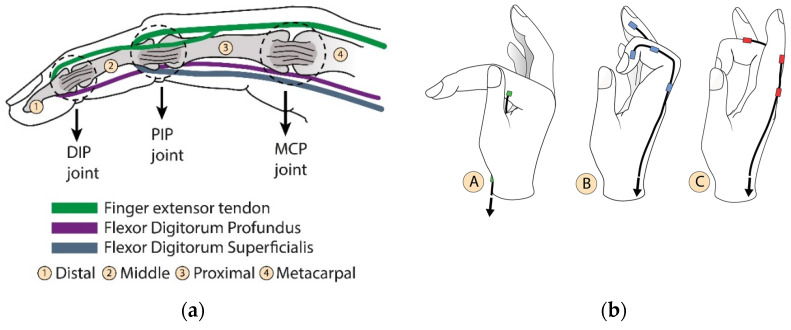

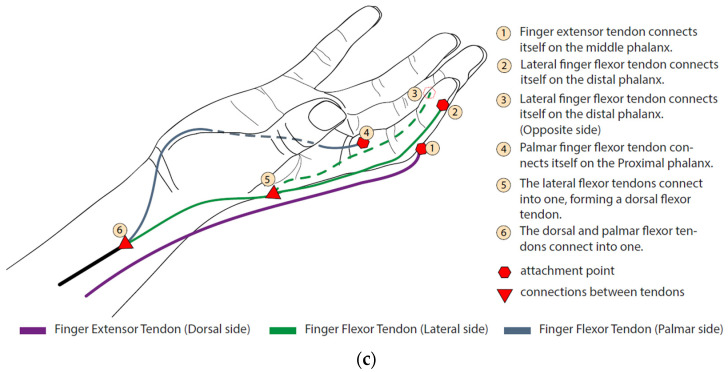
Bioinspired artificial tendons. (**a**) In green, the extensor tendon (extensor tendons) and the flexion tendons in purple and blue (digitorum profundus and digitorum superficialis, respectively). The contraction of these tendons induces angular movement of the phalanges around the DIP (distal phalanx joint), PIP (proximal phalanx joint), and MCP (metacarpal phalanx joint) articulation points. (**b**) Independent motions of the finger performed by each artificial tendon. (A) Angular movement around MCP joint; (B) finger flexion; (C) finger extension. (**c**) The index finger tendon scheme shows the distribution of the artificial extensor/flexor tendons, their fixations, and connections. This pattern is also used on the other fingers.

Figure 1a shows a biological set of the human finger composed of bones, joints, and tendons. Figure 1b describes the independent motions of the finger attributed to each artificial tendon. Different linear combinations of these movements shown at A, B, and C create narrow or wide paths for grasping and they describe 2 DoF (degree of freedom) per finger, including the thumb. Considering that the exoskeleton actuation system is designed to perform a grasping movement, a trio of artificial tendons was proposed to flex and extend the fingers, as shown in Figure 1c. Although the thumb also performs the adduction/abduction motion, the same trio of bio-inspired tendons was applied equally to each finger to replicate the kinematics of the gripping movement. An underactuated system was proposed to reduce the exoskeleton’s weight. The two tendons A and B (Figure 1b) of all fingers were pulled together using one actuator to flex the fingers. The extension movement was also carried out using one actuator. In this way, only two actuators are enough to grasp and open the hand.

**Figure 2:**
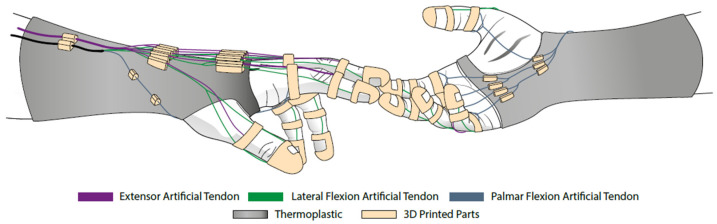
Digital prototype of the Soft Hand Exoskeleton.

One of the design goals is to allow the adaptation to different hand sizes. This objective guided the selection of exoskeleton materials, and compliant polymer-based materials were employed [27]. The hand orthosis 3D concept is shown in Figure 2. The exoskeleton is built on a semi-rigid thermoplastic orthosis, dark gray in Figure 2, which holds the wrist in a fixed position and can be accurately adjusted to different sizes of hands. The artificial tendons are anchored in the parts shown in beige tones, which were 3D manufactured via fused deposition modeling (Sethi3D S3X, Sethi3D, Campinas, Brazil), using a flexible material (Flex TPU, 3D Lab, Belo Horizonte, Brazil) to allow for adaptation to different finger diameters. The rings are placed on the fingers, whereas the anchors, placed over the thermoplastic, guide the tendons to display proper movement.

### 2.2. Hand Exoskeleton Kinematics

**Figure 3:**
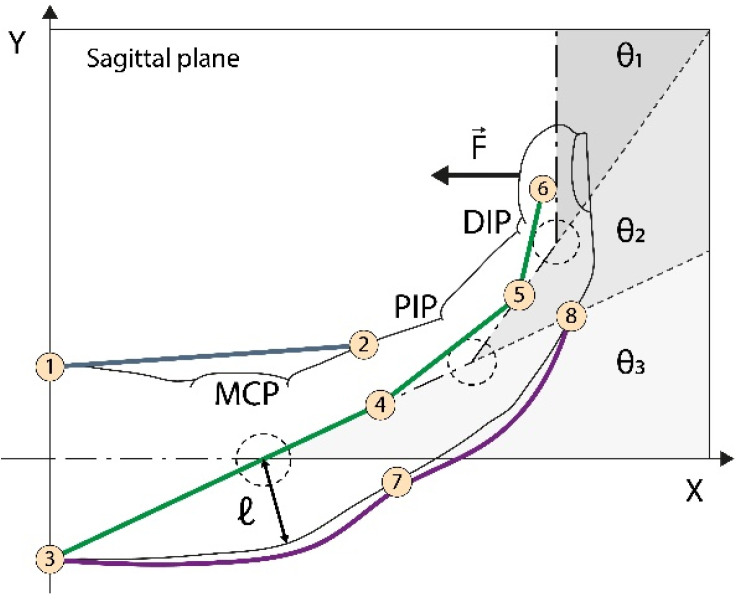
Kinematics of artificial tendons. In purple, on the back of the finger, is the tendon responsible for finger extension. The green tendon retracts the finger, while the blue one rotates the finger around the MCP (metacarpal) joint. The combined action of the blue and green tendons promotes proper flexion movement. $\theta_{1},\theta_{2},\;and\;\theta_{3}$ are the MCP, proximal interphalangeal (PIP), and distal interphalangeal DIP joint angles, respectively. l is the curvature radius reference. $P_{k}\in \left[1,8\right]$ are the wire anchor points.

The kinematic model, adapted from [28], for the hand orthosis is presented here to establish the relationship between the linear motion of the artificial tendons and the angular displacement of the finger joints. The tendon’s slack information is important in order to adjust the amount of cable pulled by the actuators, thereby optimizing the grasping time. We considered the wires to be inextensible, the longitudinal symmetry of the fingers, and we neglected friction losses. Figure 3 shows the parameters analyzed.

Figure 3 shows eight anchor points used to guide cable motion. Points $P_{1}-P_{2}$ refer to the palmar artificial tendon (PAT), $P_{3}$ to $P_{6}$ to the lateral artificial tendon (LAT), and $P_{3}-P_{7}-P_{8}$ to the extensor artificial tendon (EAT). For simplification, $P_{3}$ is used to represent two anchor points, one belonging to the EAT and the other to the LAT. The reference position is defined for $\theta_{1}=\theta_{2}=\theta_{3}=0^{\circ }$ at this position, linking the hand longitudinal symmetry line with the x -axis. The lengths are computed between the reference position and the displacement position as a function of $\theta_{i}$ .

Considering the above, the EAT wire length at the reference point ( $\delta_{r,EAT}$ ), at contract position ( $\delta_{c,EAT}$ ) and the wire length pulled by the actuator ( $\delta_{p,EAT}$ ), can be calculated as

(1) $$\delta_{r,EAT}=\bar{P_{3},P_{7}}+\bar{P_{7},P_{8}}$$

(2) $$\delta_{c,EAT}=\bar{P_{3},P_{7}}+\bar{P_{7},P_{8}}+l\left(\theta_{2}+\theta_{3}\right)$$

(3) $$\delta_{p,EAT}=l\left(\theta_{2}+\theta_{3}\right)$$

For PAT, we have the wire length at the reference point as

(4) $$\delta_{r,PAT}=\bar{P_{1},P_{2}}$$

We can use the cosine law to calculate the wire length at a contract arbitrary as

(5) $$\delta_{c,PAT}=\sqrt{{\left(\bar{P_{1},PJ_{MCP}}\right)}^{2}+{\left(\bar{P_{MCP},P_{2}}\right)}^{2}-2\left(\bar{P_{1},PJ_{MCP}}\right)\left(\bar{P_{MCP},P_{2}}\right)cos\left(\pi -\theta_{3}\right)}$$

where $Pj_{MCP}$ is the MCP point projected on the palmar side, and the wire length pulled by the actuator is the following:

(6) $$\delta_{p,PAT}=\left|\delta_{c,PAT}-\delta_{r,PAT}\right|$$

The same procedure is applied on LAT analysis:

(7) $$\delta_{r,LAT}=\bar{P_{3},P_{4}}+\bar{P_{4},P_{5}}+\bar{P_{5},P_{6}}$$

(8) $$\delta_{c,LAT}=\sum_{\zeta }\sqrt{{\left(\bar{P_{i},k}\right)}^{2}+{\left(\bar{k,P_{i+1}}\right)}^{2}-2\left(\bar{P_{i},k}\right)\left(\bar{k,P_{i+1}}\right)cos\left(\pi -\theta_{j}\right)}$$

(9) $$\delta_{p,LAT}=\left|\delta_{c,LAT}-\delta_{r,LAT}\right|$$

where $\left(i,j,k\right)\in \zeta$ , and ζ={(3,3,MCP),(4,2,PIP),(5,1,DIP)} .

**Figure 4:**
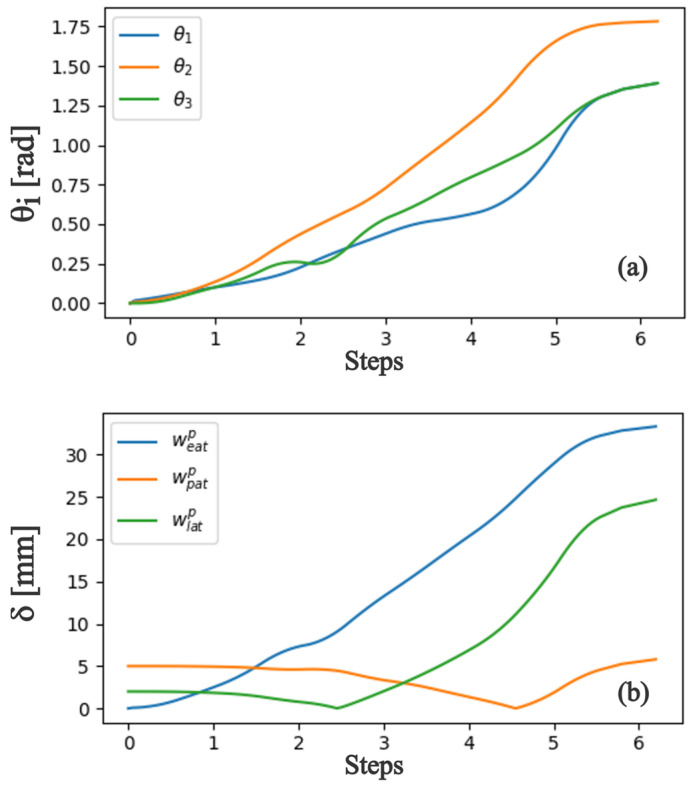
Kinematics analysis. (**a**) Angular variation: $\theta_{1}$ distal angle; $\theta_{2}$ medial angle; $\theta_{3}$ metacarpal angle. (**b**) Wire length variation: $w_{eat}^{P}$ extension length variation; $w_{pat}^{P}$ palmar length variation (rotates the finger around the MCP joint); $w_{lat}^{P}$ lateral length variation (contracts the finger).

The proposed model yields the operational parameters of angular variation of the finger joints and the artificial tendon length during the grasping movement. Figure 4a shows the expected angle variation of the joints based on data from a healthy biological hand, considering a grasping movement in six steps. This variation is used as an input to estimate the displacement of the artificial tendons (Figure 4b). This information is used as an input to develop the physical prototype of the hand exoskeleton.

### 2.3. Physical Prototype and Experimental Setup

**Figure 5:**
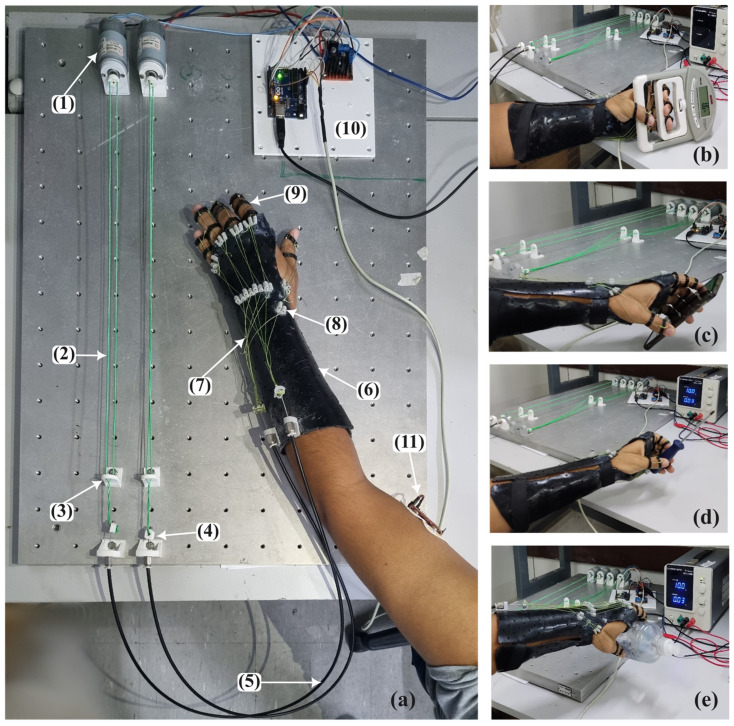
The physical prototype and experimental evaluation. (**a**) (1) DC motors. (2) Twisted string actuator cables. (3) Screen to prevent the actuator cable from tangling. (4) Actuator–conduit cable connection. (5) Bowden cables. (6) Thermoplastic orthosis. (7) Artificial tendons. (8) Addressing screen for artificial tendons. (9) Finger rings. (10) Electronics composed by Arduino Uno, and H-bridge circuit to drive the motors; (11) myoelectric sensor. (**b**) Grip strength test. (**c**) Handling a cell phone. (**d**) Handling a screwdriver. (**e**) Handling a bottle of alcohol gel.

**Figure 6:**
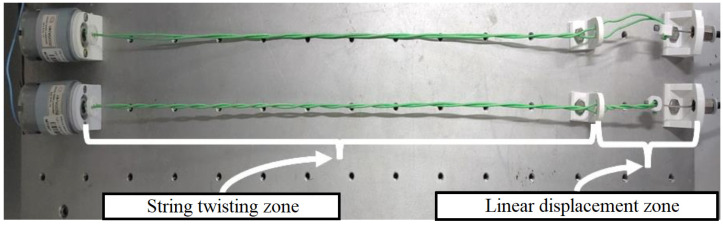
Twisted string actuator. The DC motor drives the string twisting zone, producing linear displacement of the cable.

The physical prototype of the soft hand exoskeletons and experimental setup are presented in Figure 5. The exoskeleton is composed of a black thermoplastic orthosis (6), which holds the hand in a neutral position, as shown in Figure 5a. Artificial tendons (7) are anchored in the finger rings (9), and the anchor tendons (8) are pulled by two Bowden cables (5) to perform the flexion and extension movements of the fingers. The set is driven by two micro-DC motors (12V, AK555/306PL12S6500C, Akiyama Motors, Joinville, Brazil) (1) arranged as a TSA, as shown in Figure 6. When the motors (1) are activated, the green cable (2) retracts and pulls the Bowden cable ((4) and (5)), which closes or opens the hand. When one actuator retracts the cable, the other allows the cable to elongate. This configuration simulates the extensor and flexor muscles arranged in pairs of agonists and antagonists on the forearm that open and close the hand. With the proposed design, the part of the orthosis attached to the wearer’s forearm and hand weighs just 232 g. Furthermore, the total cost to manufacture the prototype hand orthosis is between $100.00 and $150.00.

The system control hardware (10) is composed of an Arduino Uno R3 (Arduino, Italy) and an H-bridge L298N, and was designed to use the myoelectric signal of the biceps, collected using a MyoWare 2.0 Muscle Sensor (Sparkfun Eletrônics, Niwot, CO, USA) (11) to capture the user’s intention of motion to activate the motors. This approach was considered since upper-limb-impaired subjects can commonly better contract the proximal muscles of the arm, but not the distal ones. A simple but effective approach based on a custom threshold for each subject was used to identify the user’s intention to open or close the hand. Maximum voluntary isometric contraction (MVIC) was collected using the electronic setup, and a threshold corresponding to 60% of MVIC was considered to avoid muscle fatigue [29]. When the user contracts the biceps over the threshold, the system performs the opening or closing movements of the hand.

Two TSA are used to drive the exoskeleton. This type of actuation consists of winding two cables to each other to reduce their length, called the string twisting zone in Figure 6, performing a linear movement (linear displacement zone). This approach presents some advantages related to other actuators, such as compliant linear displacement; other types of reducers, which are usually heavy and bulky, are not required; high linear force output with low friction losses results in compact and high-power density actuators [25,30,31,32].

**Figure 7:**
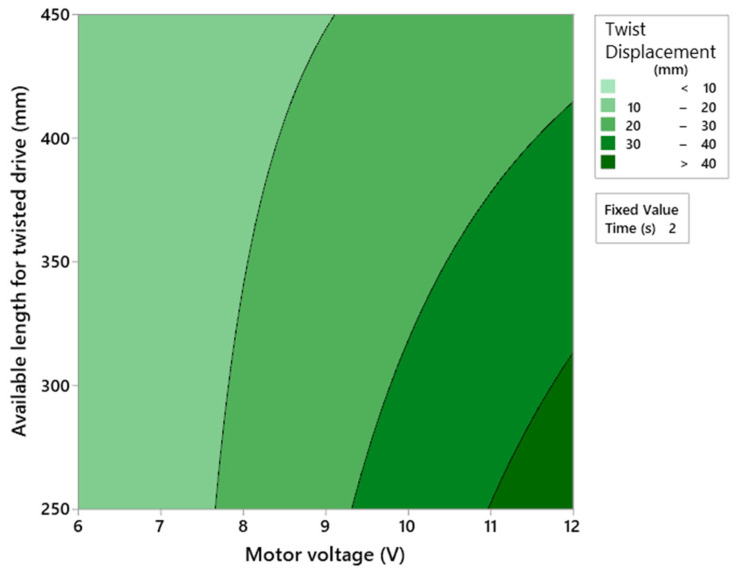
Twisted string actuation optimized parameters.

To evaluate the actuators’ performance, a 2K design of experiments was developed using three parameters: motor voltage, length of the string twisting zone, and winding time. The voltage supplied to the motor varied between 6 and 12 volts. The length available for winding (string twisting zone) varied between 250 and 450 mm. The activation time was considered to be 2 s as the time to open or close the hand. The experimental results are shown in Figure 7. For the same motor voltage, the reduced length of the string twisting zone increases the linear displacement of the cable. However, this behavior is noticed mainly because the small string twisting zone induces the cable to create a tangled spool, which can reduce the cable life. Moreover, for the same twisting zone length, increasing the motor voltage increases the linear displacement of the cable. Since, to correctly open and close the hand, a linear displacement of 20–30 mm is required (Figure 4) and to avoid cable deterioration, the string twisting zone was set at 450 mm and the motor voltage was set at 12 V.

### 2.4. Experimental Protocol and Data Analysis

Five male able-bodied subjects (85.0 ± 10.4 kg, 1.75 ± 0.04 m, and 27.4 ± 5.6 years) participated as volunteers in this study. First, the myoelectric sensor MyoWare 2.0 Muscle Sensor was placed over the biceps brachii along the longitudinal midline of the muscle, between the motor unit and the tendinous insertion of the muscle [33] after skin cleaning. Then, the subject was asked to perform three times maximum voluntary isometric contraction (MVIC) with intervals of 1 min to avoid fatigue. Overall, 60% of the mean MVIC was considered as a threshold to activate the exoskeleton, allowing the movements of opening and closing the hand. The results of the calibration section are presented in Figure S1 of the Supplementary Materials.

**Figure 8:**
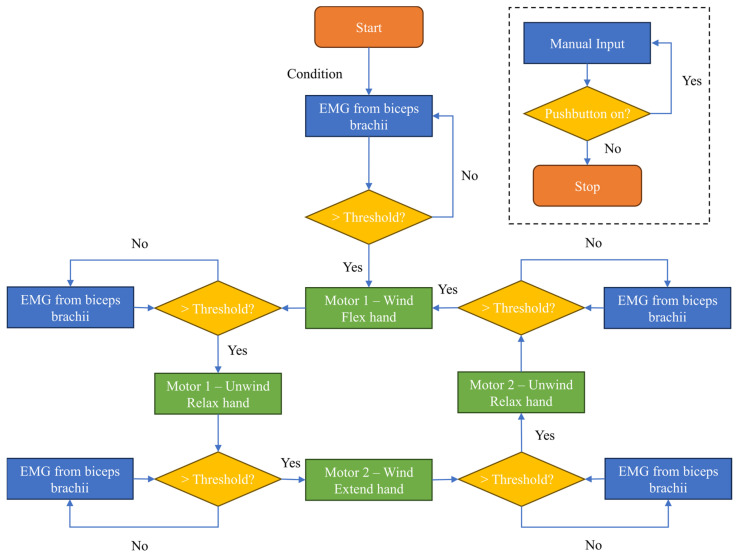
Hand orthosis operational system flowchart for the experimental protocol.

The first set of data collection was designed to train subjects to activate the system with voluntary biceps contraction and was called the training section. Ten attempts of muscle contraction were made at intervals of 30 s to avoid muscle fatigue. After the training phase, subjects were asked to simulate a paralyzed hand and try to activate the exoskeleton and manipulate three different kinds of objects: a smartphone; a screwdriver, to simulate a small object; and an alcohol gel bottle, to simulate objects with larger diameters, as shown in Figure 5c–e. Each object was manipulated three times by each subject and this set of experiments was called the testing phase. Figure 8 shows the operating system flowchart to control the hand orthosis during the testing section. The system starts with both TSA actuators in a neutral position, allowing the hand to be relaxed. A muscle contraction above the threshold (60% of the MVIC) activates motor 1, which winds the cables moving the hand in a grip position. One more muscle contraction above the threshold activates motor 1 in the opposite direction to unwind the cables and relax the hand. The same procedure is used to extend the hand after biceps contraction and relax the hand after one more muscle contraction. So, one cycle of closing and opening the hand is performed by four biceps contractions above the threshold. In this way, we ensure that the two actuators are not activated at the same time to improve safety.

We computed true positives when the subjects managed to activate the system, false negatives when the subject was not able to activate the system, and false positives when the subject involuntarily activated the system. Finally, subjects were asked to simulate a paralyzed hand, activate the exoskeleton with biceps contraction, and hold a hand dynamometer (DM-90, Instrutherm, São Paulo, Brazil) to measure grip strength, as presented in Figure 5b. The procedure was repeated three times for each subject. An extra myoelectric sensor (MyoWare 2.0 Muscle Sensor) was placed in the subject’s forearm over the flexor digitorum profundus to monitor the active flexion movement during the experiments, and no muscle activity was detected. Furthermore, since the subjects were asked to activate the system several times to avoid the fatigue of the biceps brachii during the twisting and untwisting of the cables to perform the closing and opening hand, the volunteers rested for 30 s between attempts to activate the system, and for 2 min after each set of contractions.

The performance of the system was evaluated using the accuracy metric calculated using Equation (10) and the precision metric as shown in Equation (11):

(10) $$Accuracy=\frac{\sum_{i=1}^{N}\frac{{TP}_{i}+{TN}_{i}}{{TP}_{i}+{FN}_{i}+{FP}_{i}+{TN}_{i}}}{N}$$

(11) $$Precision=\frac{\sum_{i=1}^{N}\frac{S_{i}}{S_{i}+F_{i}}}{N}$$

where *TP* are true positives, *TN* are true negatives, *FP* are false positives, and *FN* are false negatives in Equation (10) and *S* are success and *F* are failure in handling the object in Equation (11). Finally, *i* corresponds to the total attempts, and the total number of subjects is represented by *N*.

The experimental protocol was approved by the Universidade Federal do Espírito Santo Institutional Review Board (CAAE: 41368820.3.0000.5542). Subjects signed an informed consent form to participate in the study. All study procedures were carried out in accordance with all relevant guidelines and regulations.

## 3. Results and Discussion

Here, we present the results of the experimental evaluation of the hand exoskeleton in five able-bodied subjects. After calibration, the subjects were trained on how to activate the system performing biceps contraction. A total of 60% of the MVIC was used as a threshold to trigger the system, as shown in Figure 8 by the green bars. The subjects were asked to perform 10 activations with intervals of 30 s between contractions to avoid muscle fatigue. This set was named the training section. After the training section, the subjects were asked to try to grab three different kinds of objects: a smartphone, a screwdriver, and a bottle of alcohol gel. Each subject tried to handle each object three times, totaling nine attempts to trigger the system. This set of experiments was named as the testing section. In both sections, the subjects simulated a paretic hand to let just the exoskeleton orthosis move the fingers.

**Figure 9:**
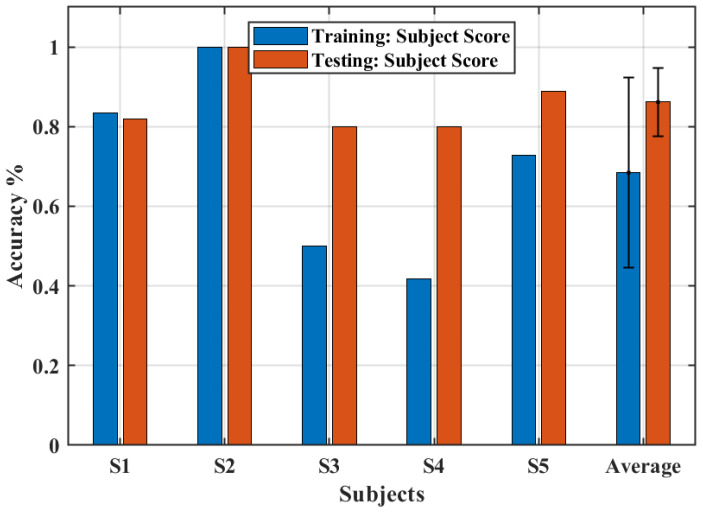
The accuracy obtained for training and test sections.

Figure 9 presents the accuracy of the system in determining the intention of the user to perform the movements of closing and opening the hand for the training section and the testing section. Whereas the average accuracy of the training section was 68.4%, the testing sections presented an average accuracy of 86.1%, which is remarkable. All subjects, except S1, had a greater accuracy after the training section. These results highlight the importance of a training section to increase the accuracy of an EMG-based trigger system [29]. Moreover, an excellent accuracy to trigger the system was obtained with a simple setup using just one myoelectric sensor.

**Figure 10:**
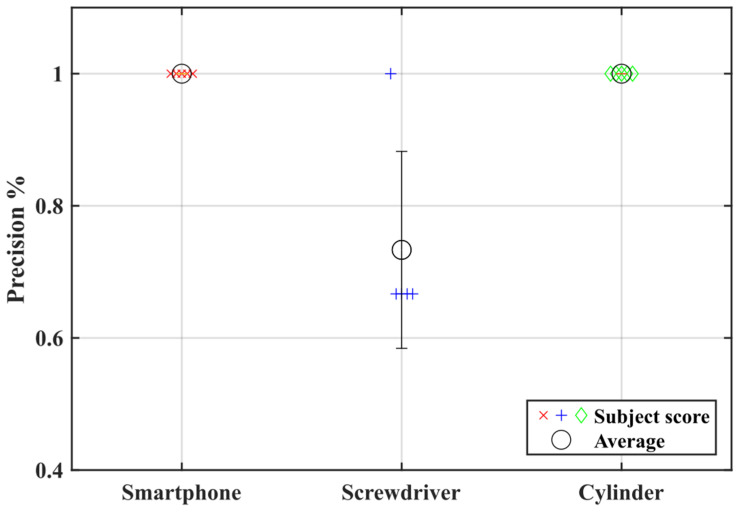
Object manipulation test. The colored symbols represent the precision score for each subject. The black circle is the average score across subjects and the error bar is the standard deviation.

The precision of handling the objects during the testing sections is shown in Figure 10. Interestingly, only the screwdriver, which represents an object of small diameter, had a precision below 100%. This outcome indicates that the proposed exoskeleton design must be improved to provide a better grasp motion for small objects. Although the biomimetic design could improve the comfort, kinematic compatibility [15,34], and tactile sensitivity of the hand, the flexion movement of the finger was limited by the designed rings, thus reducing the ability to manipulate small objects.

**Figure 11:**
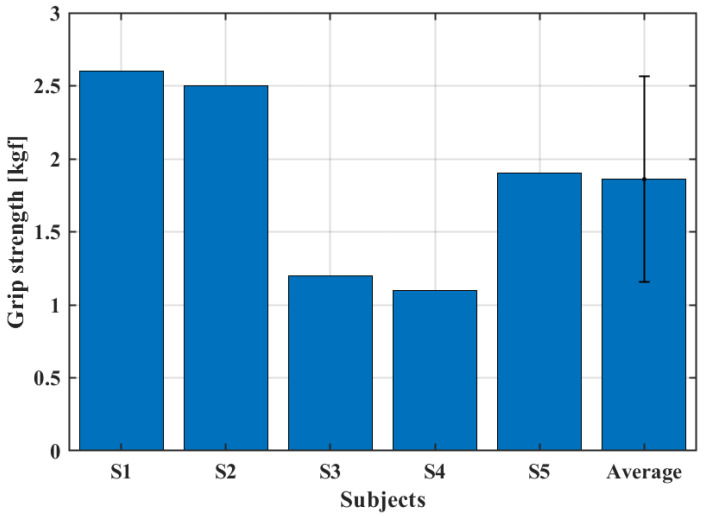
Grip strength test.

Figure 11 shows the maximum gripping force that the system could perform for each subject. In this condition, an average force of 1.86 kgf was found, which represents about 10% of the grip strength of a healthy subject [35].

At first glance, the grip strength seems to depend just on the actuators torque, since the subject was asked to not perform any contraction of the forearm muscles, simulating a paralyzed hand. However, the dimensions and shape of the hand and fingers played a role in determining the grip strength. Volunteers with larger hands displayed, on average, a greater grip strength.

## 4. Conclusions

This paper presented the biomimetic design of a soft hand exoskeleton activated by the myoelectric signal of the biceps. Three sets of artificial tendons were designed to allow the flexion and extension movements of the finger performing the hand grip. Two twisted string actuators were used to drive the artificial tendons. This configuration avoids the use of reducers to improve compactness and lightness, resulting in an orthosis attached to the user’s forearm and hand with just 232 g. The proposed design allowed for a direct interaction between the user and the object, increasing sensorial feedback by leaving the palm and finger phalanges uncovered. The accuracy and precision of the system were evaluated by five healthy subjects. The system presented an overall accuracy of 86.1% after the test section and a precision of 100% for larger objects (cellphone and alcohol gel bottle) and 73.3% for small objects (screwdriver). Moreover, the subjects were able to display an average of 1.86 kgf grip strength. The further steps of the project involve moving the actuators and control electronics into a backpack, which can be worn by the user to make the system portable. The system will be powered by a battery that can be charged by the user as needed. Moreover, we intend to implement independent movements of the fingers to allow for a better manipulation of objects. Next, experiments with people with upper-limb extremity disabilities will be carried out to evaluate the capacity of the system to restore hand functioning and increase the user’s ability to manipulate objects.

## Data Availability

Data is unavailable due to privacy or ethical restrictions.

## Supplementary Materials

The following are available online at https://www.mdpi.com/2313-7673/8/3/317/s1, Figure S1: Threshold calibration results. Maximum voluntary isometric contraction (MVIC) was performed three times (blue, orange, and yellow bars). The purple bars represent the average MVIC and the green bars represent 60% of the MVIC used as a threshold to trigger the system.

## References

[1] Padilla-Magaña J.F., Peña-Pitarch E., Sánchez-Suarez I., Ticó-Falguera N. (2022). Quantitative Assessment of Hand Function in Healthy Subjects and Post-Stroke Patients with the Action Research Arm Test. Sensors.

[2] Raghavan P. (2015). Upper Limb Motor Impairment after Stroke. Phys. Med. Rehabil. Clin..

[3] Langhorne P., Coupar F., Pollock A. (2009). Motor Recovery after Stroke: A Systematic Review. Lancet Neurol..

[4] Vos T., Abajobir A.A., Abate K.H., Abbafati C., Abbas K.M., Abd-Allah F., Abdulkader R.S., Abdulle A.M., Abebo T.A., Abera S.F. (2017). Global, Regional, and National Incidence, Prevalence, and Years Lived with Disability for 328 Diseases and Injuries for 195 Countries, 1990–2016: A Systematic Analysis for the Global Burden of Disease Study 2016. Lancet.

[5] Feigin V.L., Stark B.A., Johnson C.O., Roth G.A., Bisignano C., Abady G.G., Abbasifard M., Abbasi-Kangevari M., Abd-Allah F., Abedi V. (2021). Global, Regional, and National Burden of Stroke and Its Risk Factors, 1990–2019: A Systematic Analysis for the Global Burden of Disease Study 2019. Lancet Neurol..

[6] Hatem S.M., Saussez G., della Faille M., Prist V., Zhang X., Dispa D., Bleyenheuft Y. (2016). Rehabilitation of Motor Function after Stroke: A Multiple Systematic Review Focused on Techniques to Stimulate Upper Extremity Recovery. Front. Hum. Neurosci..

[7] Lang C.E., MacDonald J.R., Reisman D.S., Boyd L., Kimberley T.J., Schindler-Ivens S.M., Hornby T.G., Ross S.A., Scheets P.L. (2009). Observation of Amounts of Movement Practice Provided During Stroke Rehabilitation. Arch. Phys. Med. Rehabil..

[8] Waddell K.J., Birkenmeier R.L., Moore J.L., Hornby T.G., Lang C.E. (2014). Feasibility of High-Repetition, Task-Specific Training for Individuals with Upper-Extremity Paresis. Am. J. Occup. Ther..

[9] Kleim J.A., Jones T.A. (2008). Principles of Experience-Dependent Neural Plasticity: Implications for Rehabilitation After Brain Damage. J. Speech Lang. Hear. Res..

[10] Bertani R., Melegari C., De Cola M.C., Bramanti A., Bramanti P., Calabrò R.S. (2017). Effects of Robot-Assisted Upper Limb Rehabilitation in Stroke Patients: A Systematic Review with Meta-Analysis. Neurol. Sci..

[11] Heo P., Gu G.M., Lee S., Rhee K., Kim J. (2012). Current Hand Exoskeleton Technologies for Rehabilitation and Assistive Engineering. Int. J. Precis. Eng. Manuf..

[12] Leonardis D., Barsotti M., Loconsole C., Solazzi M., Troncossi M., Mazzotti C., Castelli V.P., Procopio C., Lamola G., Chisari C. (2015). An EMG-Controlled Robotic Hand Exoskeleton for Bilateral Rehabilitation. IEEE Trans. Haptics.

[13] Gasser B.W., Bennett D.A., Durrough C.M., Goldfarb M. Design and Preliminary Assessment of Vanderbilt Hand Exoskeleton. Proceedings of the 2017 International Conference on Rehabilitation Robotics (ICORR).

[14] Andrade R.M., Bonato P. (2021). The Role Played by Mass, Friction, and Inertia on the Driving Torques of Lower-Limb Gait Training Exoskeletons. IEEE Trans. Med. Robot. Bionics.

[15] de Andrade R.M., Fabriz Ulhoa P.H., Fragoso Dias E.A., Filho A.B., Vimieiro C.B.S. (2023). Design and testing a highly backdrivable and kinematic compatible magneto-rheological knee exoskeleton. J. Intell. Mater. Syst. Struct..

[16] Noronha B., Accoto D. (2021). Exoskeletal Devices for Hand Assistance and Rehabilitation: A Comprehensive Analysis of State-of-the-Art Technologies. IEEE Trans. Med. Robot. Bionics.

[17] Novelli G.L., Vargas G.G., Andrade R.M. (2023). Dielectric elastomer actuators as artificial muscles for wearable robots. J. Intell. Mater. Syst. Struct..

[18] Pinskier J., Shirinzadeh B. Topology optimization of leaf flexures for stiffness ratio maximization in compliant mechanisms. Proceedings of the 2018 IEEE/ASME International Conference on Advanced Intelligent Mechatronics (AIM).

[19] Sun Y., Lueth T.C. (2023). Enhancing Torsional Stiffness of Continuum Robots Using 3-D Topology Optimized Flexure Joints. IEEE/ASME Trans. Mechatron..

[20] In H., Kang B.B., Sin M., Cho K.-J. (2015). Exo-Glove: A Wearable Robot for the Hand with a Soft Tendon Routing System. IEEE Robot. Autom. Mag..

[21] Nycz C.J., Bützer T., Lambercy O., Arata J., Fischer G.S., Gassert R. (2016). Design and Characterization of a Lightweight and Fully Portable Remote Actuation System for Use with a Hand Exoskeleton. IEEE Robot. Autom. Lett..

[22] Xiloyannis M., Cappello L., Khanh D.B., Yen S.-C., Masia L. Modelling and Design of a Synergy-Based Actuator for a Tendon-Driven Soft Robotic Glove. Proceedings of the 2016 6th IEEE International Conference on Biomedical Robotics and Biomechatronics (BioRob).

[23] Palli G., Natale C., May C., Melchiorri C., Wurtz T. (2013). Modeling and Control of the Twisted String Actuation System. IEEE/ASME Trans. Mechatron..

[24] Würtz T., May C., Holz B., Natale C., Palli G., Melchiorri C. The Twisted String Actuation System: Modeling and Control. Proceedings of the 2010 IEEE/ASME International Conference on Advanced Intelligent Mechatronics.

[25] Hosseini M., Sengül A., Pane Y., De Schutter J., Bruyninck H. ExoTen-Glove: A Force-Feedback Haptic Glove Based on Twisted String Actuation System. Proceedings of the 2018 27th IEEE International Symposium on Robot and Human Interactive Communication (RO-MAN).

[26] Standring S. (2015). Gray’s Anatomy E-Book: The Anatomical Basis of Clinical Practice.

[27] Cornejo J., Cornejo-Aguilar J.A., Vargas M., Helguero C.G., De Andrade R.M., Torres-Montoya S., Asensio-Salazar J., Rivero Calle A., Martínez Santos J., Damon A. (2022). Anatomical Engineering and 3D Printing for Surgery and Medical Devices: International Review and Future Exponential Innovations. BioMed Res. Int..

[28] Lourenço B., Neto V., Andrade R.d. (2020). A Concept Design of an Adaptive Tendon Driven Mechanism for Active Soft Hand Orthosis. Proceedings.

[29] Lorrain T., Jiang N., Farina D. (2011). Influence of the training set on the accuracy of surface EMG classification in dynamic contractions for the control of multifunction prostheses. J. NeuroEngineering Rehabil..

[30] Suthar B., Usman M., Seong H., Gaponov I., Ryu J. Preliminary Study of Twisted String Actuation Through a Conduit Toward Soft and Wearable Actuation. Proceedings of the IEEE International Conference on Robotics and Automation (ICRA).

[31] Jeong S.H., Kim K., Kim S. (2017). Designing Anthropomorphic Robot Hand with Active Dual-Mode Twisted String Actuation Mechanism and Tiny Tension Sensors. IEEE Robot. Autom. Lett..

[32] Rúbio G.d.P., Martins Ferreira F.M.R., Brandão F.H.d.L., Machado V.F., Tonelli L.G., Martins J.S.R., Kozan R.F., Vimieiro C.B.S. (2020). Evaluation of Commercial Ropes Applied as Artificial Tendons in Robotic Rehabilitation Orthoses. Appl. Sci..

[33] De Luca C.J. (2002). Surface Electromyography: Detection and Recording.

[34] Fiorezi G.G., dos Santos de Moraes J., Ulhoa P.H.F., de Andrade R.M. (2020). Biomimetic design of a planar torsional spring to an active knee prosthesis actuator using FEM analysis. Proceedings.

[35] Massy-Westropp N.M., Gill T.K., Taylor A.W., Bohannon R.W., Hill C.L. (2011). Hand Grip Strength: Age and gender stratified normative data in a population-based study. BMC Res. Notes.

