# Supplementary material for: Biomimetic Design of a Tendon-Driven Myoelectric Soft Hand Exoskeleton for Upper-Limb Rehabilitation
Source: Biomimetics (Basel). 2023 Jul 19;8(3):317. doi: 10.3390/biomimetics8030317 (PMC10807486; doi:10.3390/biomimetics8030317)
Supplement: Supplementary file 1 [file biomimetics-08-00317-s001.zip › biomimetics-2509963-supplementary.pdf]

Supplementary Material

Biomimetic Design of a Tendon-Driven Myoelectric Soft Hand Exoskeleton for Upper-Limb Rehabilitation

The results of the calibration phase are shown in Figure S.1. Each subject performed MVIC of the biceps, where the myoelectric sensors were placed, three times (blue, orange, and yellow bars). The muscle contractions of each volunteer were very different, and volunteer 2 reached the maximum muscle activity, as measured by the sensor (970).

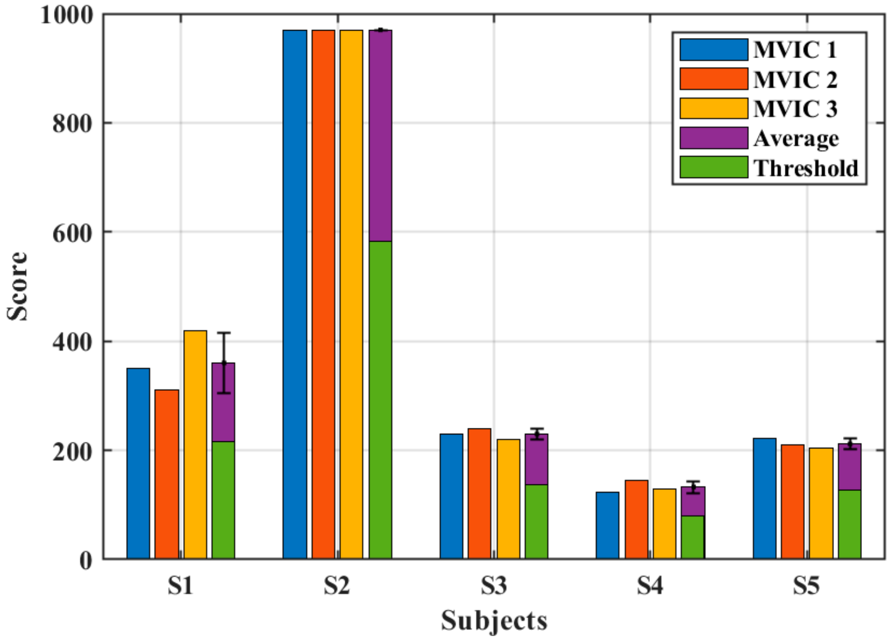

Figure S.1. Threshold calibration results. Maximum voluntary isometric contraction (MVIC) was performed three times (blue, orange, and yellow bars). The purple bars represent the average MVIC and the green bars represent 60% of the MVIC used as a threshold to trigger the system.
